# Social Determinants of Quality of Life in the Last Year of Life Among Older Adults: A Comparative Mixed Methods Study

**DOI:** 10.1093/geroni/igaf122.1700

**Published:** 2025-12-31

**Authors:** Raeann LeBlanc, Sangduan Ginggeaw, Joohyun Chung

**Affiliations:** University of Massachusetts Amherst, Amherst, Massachusetts, United States; University of Massachusetts Amherst, Amherst, Massachusetts, United States; University of Massachusetts Amherst, Amherst, Massachusetts, United States

## Abstract

Quality of life (QOL) in the last year of life declines among community-dwelling older adults with multimorbidity, with social determinants playing a critical role. However, social capital remains underexamined in nursing research, particularly in end-of-life care. This study explores how social determinants, especially social capital, influence QOL in this population. A convergent mixed-methods design was used. The quantitative component analyzed data from 230 participants in the National Health and Aging Trends Study (NHATS) using linear regression and path analysis to examine social capital’s mediating role between social determinants and QOL. The qualitative component involved thematic analysis of semi-structured interviews with seven family caregivers. Findings were integrated through joint displays and meta-inferences. Results showed that QOL in the last year of life was generally low, as was social capital. Mental conditions and social capital were key predictors of QOL, with social capital partially mediating the relationship between mental conditions and QOL. Thematic analysis identified six key themes: financial well-being, access to resources through social networks, supportive relationships, acceptance of death, positive emotions, and manageable physical symptoms. Meta-inferences revealed that mental health and social capital significantly influence QOL. Social capital not only mediates the relationship between mental conditions and QOL but also extends to socioeconomic factors, physical symptoms and disability. Family caregivers, acting as bridging social capital, connect patients to broader support networks, positively impacting QOL and highlighting the need for care strategies that enhance social capital to improve end-of-life QOL for both patients and caregivers.

